# Psychometric evaluation of the Korean version of the Child Oral Health Impact Profile Short Form (COHIP-SF 19): a cross-sectional study

**DOI:** 10.1186/s12955-026-02540-w

**Published:** 2026-05-09

**Authors:** Ji-Soo Song

**Affiliations:** Department of Pediatric Dentistry, Dental Research Institute, School of Dentistry, Seoul National University, 101 Daehak-ro, Jongno-gu, Seoul, 03080 Republic of Korea

**Keywords:** Oral health-related quality of life, COHIP-SF 19, Factor analysis, Reliability, Validity, School children, Korean

## Abstract

**Background:**

The Child Oral Health Impact Profile Short Form 19 (COHIP-SF 19) is widely used to assess oral health-related quality of life (OHRQoL). However, its psychometric properties have not been evaluated in Korea. This study aimed to examine the factor structure of the Korean COHIP-SF 19 and to assess its reliability and validity in schoolchildren.

**Methods:**

Data were obtained from 980 fourth-grade elementary school students (aged 10 years) participating in a 2023 national oral health program. Children completed the COHIP-SF 19 online, and clinical examinations recorded caries, malocclusion, gingivitis, and oral hygiene status. Exploratory and confirmatory factor analyses were performed. Reliability was assessed using Cronbach’s alpha, corrected item-total correlations, and test–retest intraclass correlation coefficients (ICC). Convergent and discriminant validity were evaluated using self-rated oral health, perceived treatment need, and clinical findings.

**Results:**

Exploratory factor analysis supported a four-factor solution with separation of the self-image subscale. Confirmatory factor analysis showed better fit for the modified model than the original structure. The total score demonstrated excellent test–retest reliability (ICC = 0.905). Cronbach’s alpha for the 19 items was 0.729, with higher values in the modified model. Convergent validity was confirmed by correlations with self-rated oral health and treatment need. Discriminant validity was supported: children with caries or malocclusion had lower scores, whereas gingivitis and oral hygiene showed no associations. Gender differences appeared only in the modified model, with girls reporting lower functional well-being and social well-being scores.

**Conclusions:**

The Korean COHIP-SF 19 demonstrated satisfactory psychometric properties in 10-year-old schoolchildren. The modified model showed improved structural validity, higher internal consistency, and better subgroup discrimination, including by gender. These findings support its use in clinical and epidemiological research.

## Background

Oral health is closely linked to the overall health and quality of life of children and adolescents; however, clinical indicators alone cannot fully capture their perceptions, functional limitations, or psychosocial experiences [1, 2]. To address this limitation, several multidimensional instruments have been developed to measure oral health-related quality of life (OHRQoL), reflecting the distinct developmental, psychosocial, and disease-related characteristics that characterize pediatric populations [3, 4]. Among these instruments, the Child Oral Health Impact Profile (COHIP) is one of the most widely used [5]. The original instrument consists of 34 items (COHIP-34) and has been cross-culturally adapted and validated in numerous countries, including Korean children aged 8–15 years [6, 7].

From a clinical perspective, OHRQoL measures assist clinicians in understanding patients’ conditions and making informed treatment decisions while helping patients recognize their treatment needs and interpret outcomes [8]. These instruments are also widely applied in epidemiological research to evaluate oral health care needs in general and vulnerable populations [9]. However, the length of the COHIP-34 imposes a considerable response burden in pediatric clinical and research settings [10]. To address this issue while retaining the core domains of the original instrument, the 19-item short form (COHIP-SF 19) was developed in 2012 as a more concise measure of OHRQoL [8].

The COHIP-SF 19 has been translated and validated in various cultural settings, including Japanese, Chinese, Portuguese, Arabic, French, and German populations, yet no validated Korean version currently exists [9, 11–15]. Furthermore, the reproducibility of its factor structure across cultures remains controversial. Although the original COHIP-SF 19 consists of three subscales–oral health (OH), functional well-being (FW), and social-emotional well-being (SW)–previous studies have reported low factor loadings for certain items and indistinct boundaries between subscales in diverse research contexts [9, 11]. These observations highlight the importance of examining the instrument’s structural validity through exploratory and confirmatory factor analyses (EFA and CFA) and, where indicated, proposing revised models that better suit Korean children.

Accordingly, the present study had two objectives. The first was to investigate the factor structure of the Korean COHIP-SF 19 in children and adolescents using EFA and CFA and to determine the most appropriate model for this population. The second was to assess the reliability, convergent validity, and discriminant validity of both the original and the proposed models, thereby establishing an evidence-based rationale for the clinical and epidemiological use of the COHIP-SF 19 in Korea.

## Methods

### Study population

This study was conducted within a larger project evaluating OHRQoL among participants in the Student Dental Home Program (SDHP), a major oral health promotion initiative that provides preventive, community-based dental services without charge to all fourth-grade students in Seoul, who are typically 10 years old [16]. After parental registration via a mobile application, children attend designated dental clinics for medical history review, oral examination, and individualized counseling on oral hygiene, diet, and fluoride use. Preventive procedures, including professional tooth cleaning, fluoride application, sealant placement, and scaling, are also delivered during the same visit. The 2023 SDHP ran from May to November and encompassed 23,937 eligible students. Study information was distributed between July and September.

### Survey procedure

When parents opened the SDHP mobile application, a banner and pop-up notification informed them about the survey. Selecting either option redirected them to a Google Form, where the first page contained a detailed study description. Students then completed the questionnaire themselves, reporting their oral health experiences over the previous three months. To assess test–retest reliability, all participants were invited to complete the identical questionnaire again after one week. Those who finished both assessments received a mobile gift certificate worth approximately USD 8.

### Ethics approval and consent to participate

This study was approved by the Institutional Review Board of Seoul National University School of Dentistry (IRB No. S-D20190018). Informed consent from both parent and child was required before continuing.

### COHIP-SF 19 questionnaire

The Korean COHIP-SF 19 was constructed by extracting the 19 items recommended by Broder et al. from the previously validated Korean COHIP-34 [6–8]. In the original structure, the items load onto three domains–OH, FW, and SW–which comprise five, four, and ten items, respectively. Item content is shown in Table 1.

**Table 1:** Item-level response distribution and descriptive statistics of COHIP-SF 19

Items		Negative impacts	Neutral	Positive impacts	Score	
		N (%)			Median (range)	Mean (SD)
Total 19 items		705 (71.9)				
Negatively worded items		224 (22.9)				
Positively worded items		648 (66.1)				
OH1	Toothache	8 (0.8)	101 (10.3)	871 (88.9)	4 (1–4)	3.5 (0.7)
OH3	Discolored teeth	33 (3.4)	110 (11.2)	837 (85.4)	4 (0–4)	3.5 (0.9)
OH4	Crooked teeth or spaces between teeth	87 (8.9)	92 (9.4)	801 (81.7)	4 (0–4)	3.3 (1.1)
OH6	Bad breath	117 (11.9)	312 (31.8)	551 (56.2)	3 (0–4)	2.7 (1.0)
OH7	Bleeding gums	16 (1.6)	78 (8.0)	886 (90.4)	4 (0–4)	3.6 (0.7)
FW2	Difficulty eating	9 (0.9)	14 (1.4)	957 (97.7)	4 (0–4)	3.8 (0.5)
FW3	Trouble sleeping	2 (0.2)	3 (0.3)	975 (99.5)	4 (0–4)	3.9 (0.3)
FW4	Difficulty pronouncing	2 (0.2)	11 (1.1)	967 (98.7)	4 (1–4)	3.9 (0.3)
FW6	Difficulty keeping teeth clean	13 (1.3)	35 (3.6)	932 (95.1)	4 (0–4)	3.7 (0.6)
SW1	Unhappy or sad	6 (0.6)	36 (3.7)	938 (95.7)	4 (0–4)	3.8 (0.5)
SW2	Felt worried or anxious	6 (0.6)	70 (7.1)	904 (92.2)	4 (1–4)	3.6 (0.6)
SW3	Avoided smiling	3 (0.3)	29 (3.0)	948 (96.7)	4 (1–4)	3.9 (0.4)
SW4	Felt looked different	7 (0.7)	32 (3.3)	941 (96.0)	4 (1–4)	3.8 (0.5)
SW5	Worried about other’s thinking about teeth/face	11 (1.1)	47 (4.8)	922 (94.1)	4 (0–4)	3.7 (0.6)
SW7	Been teased	0 (0.0)	4 (0.4)	976 (99.6)	4 (2–4)	4.0 (0.2)
SE1	Missed school	0 (0.0)	3 (0.3)	977 (99.7)	4 (2–4)	4.0 (0.2)
SE3	Not wanted to speak	0 (0.0)	2 (0.2)	978 (99.8)	4 (2–4)	4.0 (0.2)
SI1	Been confident	498 (50.8)	237 (24.2)	245 (25.0)	1 (0–4)	1.4 (1.3)
SI2	Felt being attractive	603 (61.5)	238 (24.3)	139 (14.2)	1 (0–4)	1.1 (1.2)

For negatively worded items: Negative impacts = Almost all the time (0) or quite often (1); Neutral = Sometimes (2); Positive impacts = Almost never (3) or never (4). For positively worded items: Negative impacts = Strongly disagree (0) or somewhat disagree (1); Neutral = Neither agree nor disagree (2); Positive impacts = Somewhat agree (3) or strongly agree (4). OH, oral health; FW, functional well-being; SW, social/emotional well-being; SE, school environment; SI, self-image

Each item was scored on a 5-point Likert scale (0–4). Negatively worded items were rated from 0 (“almost always”) to 4 (“never”), whereas the two positively worded SI items were reverse-coded. Higher scores reflect better OHRQoL. Subscale scores were obtained by summing the respective items, and the total score was the sum of all 19 items. Responses of 0 or 1 were considered to indicate a negative impact.

### External criteria for validity

Participants also rated their perceived overall oral health and perceived need for dental treatment on a 6-point Likert scale ranging from 0 (“very poor” or “not needed at all”) to 5 (“very good” or “strongly needed”).

Clinical data from the SDHP included caries experience (dft/DMFT), malocclusion, gingivitis, and oral hygiene status. Teeth with an International Caries Detection and Assessment System score ≥ 4 were classified as carious [17]. Malocclusion was defined as a Dental Health Component grade ≥ 3 on the Index of Orthodontic Treatment Need [18]. Gingivitis was recorded when bleeding, swelling, or calculus was present. Oral hygiene was evaluated using the Patient Hygiene Performance index and categorized as good (0–10), fair (11–20), or poor (21–30) [19].

### Statistical analysis

Following COSMIN guidelines, structural validity, internal consistency, test–retest reliability, convergent validity, and discriminant validity of the Korean COHIP-SF 19 were examined [20]. Analyses were conducted using IBM SPSS Statistics version 31.0 and AMOS version 31.0 (IBM Corp., Armonk, NY, USA). Statistical significance was set at *p* < 0.05.

#### Factor analysis

EFA was performed to investigate the latent structure of the Korean COHIP-SF 19. All 19 items were entered into principal axis factoring with Promax rotation (Kaiser normalization). Data suitability was confirmed by the Kaiser–Meyer–Olkin (KMO) measure and Bartlett’s test of sphericity.

CFA was first conducted on the original three-subscale model, followed by alternative models derived from EFA findings. Maximum likelihood estimation was used. Model fit was assessed using the chi-square/degrees of freedom ratio (χ^2^/df), Comparative Fit Index (CFI), Tucker–Lewis Index (TLI), Root Mean Square Error of Approximation (RMSEA), and Standardized Root Mean Square Residual (SRMR). Model comparisons relied on the Akaike Information Criterion (AIC). Acceptable fit was defined as χ^2^/df ≤ 3, CFI and TLI ≥ 0.90, RMSEA ≤ 0.08, and SRMR ≤ 0.10; lower AIC values indicated superior comparative fit [21].

Modification indices (MI) were inspected for potential improvements. Error covariances were added only when modification index values exceeded 30, when items loaded on the same factor, and when theoretically justified. Fit changes were evaluated after each modification. Items with standardized factor loadings below 0.40 were reviewed for potential removal.

A second-order model was tested to determine whether the final first-order structure (including retained error covariances) could be explained by a single higher-order OHRQoL factor; fit indices were compared accordingly.

#### Reliability

Test–retest reliability was evaluated using intraclass correlation coefficients (ICC) based on a two-way random-effects model with absolute agreement. Internal consistency of the total scale and each subscale was assessed using Cronbach’s α, including α-if-item-deleted values for both original and modified models. Coefficients ≥ 0.70 were deemed acceptable [22].

Scale homogeneity was examined through corrected item-total and item-subscale correlations (Spearman ρ), computed after removing the respective item from the total or subscale score [23]. Values ≥ 0.20 were considered sufficient for item retention [24].

#### Validity

Convergent validity was determined by Spearman correlations between COHIP-SF 19 scores and self-rated overall oral health and perceived treatment need, reported for both the original and final models. Discriminant validity was tested by comparing COHIP-SF 19 scores across clinical groups (caries experience, malocclusion, gingivitis, oral hygiene status) and sex using Mann–Whitney U tests or Kruskal–Wallis tests, as appropriate.

## Results

### Participants’ demographic characteristics

Of the 20,495 students enrolled in the 2023 Student Dental Home Program, 1,068 (5.2%) completed both the baseline and one-week follow-up questionnaires. Forty-two participants who responded multiple times were retained only once (first submission), and 46 who completed the questionnaire without attending the clinical examination were excluded. The final sample comprised 980 children (4.78% of eligible students). All participants were Korean, resided in Seoul, and came from all 25 administrative districts and 166 of the 608 elementary schools. Mean age was 10.1 ± 0.4 years, and the sample included 508 boys (51.8%) and 472 girls (48.2%), with no significant age difference by sex. ICC was calculated by comparing responses from the baseline and follow-up questionnaires, whereas all other analyses were conducted using baseline data.

### Item-level response distribution

Item-level score distributions are shown in Table 1. Overall, 71.9% of participants reported at least one negative impact across the 19 items. Among the 17 negatively worded items, 22.9% reported at least one negative impact, whereas 66.1% did so on the two positively worded items. All items belonging to the SI domain exhibited high rates of negative impact (>50%). Apart from the SI subscale, only item OH6 showed a noteworthy frequency of negative responses (11.9%); the remaining items had very low rates.

### Factor analysis

EFA confirmed data suitability (KMO = 0.812; Bartlett’s test: χ^2^ (171) = 4,232.789, *p* < 0.001). Although six factors initially emerged, several contained too few items and displayed domain fragmentation. Three- and four-factor solutions were therefore examined based on the scree plot (Table 2). Across solutions, the two SI items consistently formed a separate factor. Item FW4 loaded on the SW factor, and item OH1 showed cross-loadings on both OH and FW factors.

**Table 2:** Exploratory factor analysis of Korean version of COHIP-SF 19

	EFA component												
	Initial (Unrestricted; EV > 1)						Fixed 3-Factor Solution			Fixed 4-Factor Solution			
	1	2	3	4	5	6	1	2	3	1	2	3	4
OH1		0.371				0.202		0.218			**0.565**		
OH3				**0.770**				**0.684**					**0.699**
OH4				**0.459**				**0.512**					**0.508**
OH6				0.280				**0.453**					0.373
OH7						**0.711**		0.394			0.315		
FW2		**0.812**					0.358				**0.600**		
FW3		**0.486**					0.379				**0.434**		
FW4	0.274						0.351			0.273			
FW6		**0.463**					0.282	0.234			**0.516**		
SW1		0.264			**0.459**		**0.682**			0.356	**0.507**		
SW2					**0.842**		**0.620**			**0.416**	0.316		
SW3	**0.572**						**0.655**			**0.658**			
SW4	**0.888**						**0.714**			**0.829**			
SW5	**0.720**						**0.633**			**0.786**			
SW7	**0.435**						**0.420**			**0.456**			
SE1													
SE3	0.342						0.350			0.334			
SI1			**0.816**						**0.809**			**0.815**	
SI2			**0.857**						**0.856**			**0.853**	

Only factor loadings ≥ 0.20 from the pattern matrix are displayed; loadings ≥ 0.40 are highlighted in bold

OH, oral health; FW, functional well-being; SW, social/emotional well-being; SE, school environment; SI, self-image

**Fig. 1:**
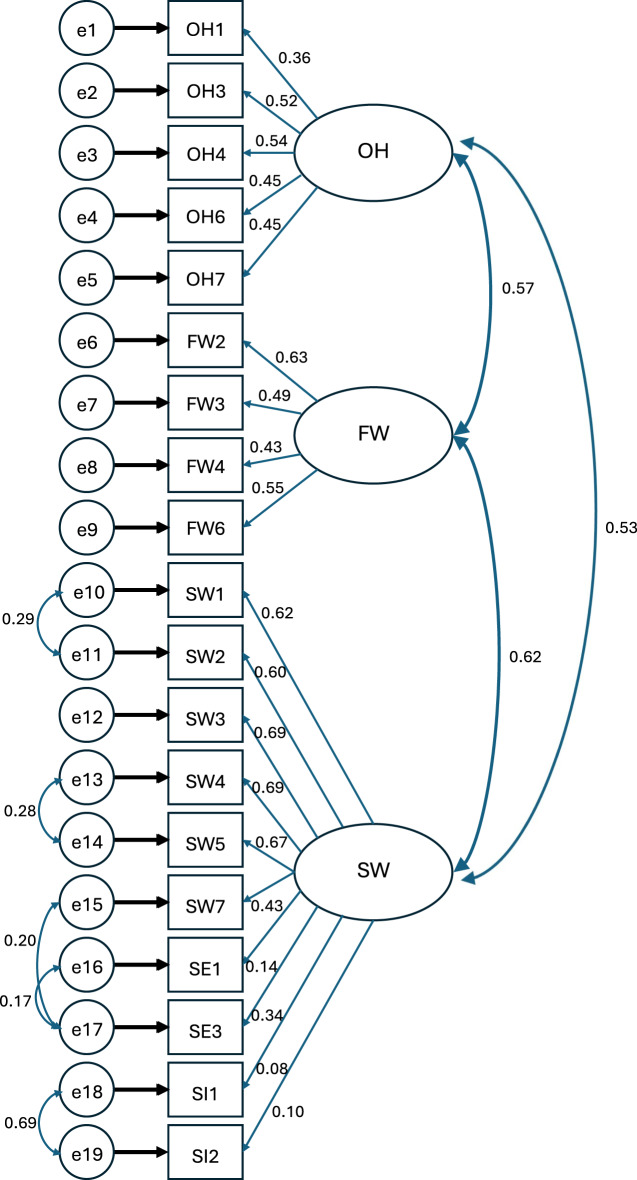
Confirmatory factor analysis of COHIP-SF 19 with the original three-subscale structure (Model 1)

The CFA results are summarized in Table 3. An initial CFA based on the original three-subscale structure of the COHIP-SF 19 was conducted (Model 1). Error covariances were added for five item pairs with high MI (≥30); all pairs were located within the SW domain of the original structure and were conceptually related (Model 1, Version 3). However, model fit remained at the borderline of acceptable thresholds. Factor correlations ranged from 0.53 to 0.62, and items SI1 and SI2 showed very low factor loadings (≤0.10) (Fig. 1).

**Table 3:** Model fit indices from confirmatory factor analysis of the Korean version of COHIP-SF 19

Model	Version	χ^2^/df	CFI	TLI	RMSEA	SRMR	AIC	High MI values	
1	1	9.720	0.683	0.636	0.094	0.078	1,568.3	SI1–SI2	471.2
	2	5.456	0.839	0.814	0.067	0.060	929.5	SW1–SW2	82.91
								SW4–SW5	53.87
								SW7–SE3	42.86
								SE1–SE3	32.48
	3	4.180	0.888	0.867	0.057	0.055	731.9	No items with MI > 30	
2	1	5.461	0.841	0.814	0.068	0.059	885.3	SW1–SW2	82.70
								SW4–SW5	54.47
								SW7–SE3	42.77
								SE1–SE3	32.49
	2	4.164	0.890	0.868	0.057	0.054	687.3	No items with MI > 30	
3	1	4.799	0.864	0.841	0.062	0.054	788.6	SW1–SW2	86.06
								SW4–SW5	54.63
								SW7–SE3	41.49
								SE1–SE3	31.73
	2† (final model)	3.467	0.914	0.897	0.050	0.049	588.3	No items with MI > 30	
4	1	4.545	0.895	0.874	0.060	0.051	627.6	SW1–SW2	80.71
								SW4–SW5	53.34
	2	3.440	0.929	0.913	0.050	0.048	499.8	No items with MI > 30	
Second-order		3.481	0.913	0.896	0.050	0.050	593.2		

Model 1: Original three-subscale structure; Model 2: Four-subscale structure with the Self-Image subscale separated; Model 3: Final model. Modified four-subscale structure based on Model 2, with item OH1 moved to the Functional Well-being subscale and item FW4 moved to the Socio-emotional Well-being subscale; Model 4: Model 3 with SE1 and SE3 (factor loadings < 0.40) removed; Second-order model: A higher-order COHIP factor added to Model 3 (Version 2), retaining all first-order factor loadings and error covariances. Version 2 and Version 3 represent stepwise modifications wherein correlated error terms were added between item pairs within the same subscale when the modification index exceeded 30 in the preceding version. χ^2^/df, chi-square minimum discrepancy divided by degrees of freedom; CFI, comparative fit index; TLI, Tucker–Lewis index; RMSEA, root mean square error of approximation; SRMR, standardized root mean square residual; AIC, Akaike information criterion; MI, modification index

**Fig. 2:**
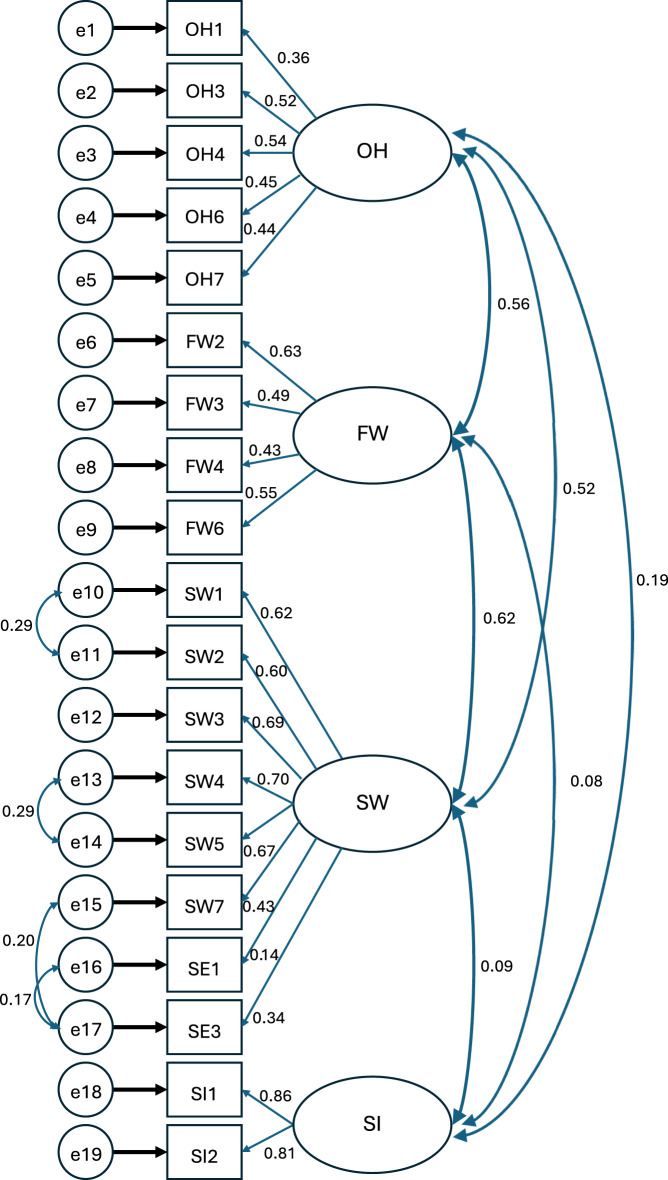
Confirmatory factor analysis of COHIP-SF 19 with the modified four-subscale structure (Model 2). In this model, self-image items are separated from the Social/Emotional well-being domain of model 1

To reflect the differentiation in the EFA, a four-factor model that separated SI as an independent domain was tested (Model 2). SI1 and SI2 were strongly correlated (Pearson *r* = 0.696, *p* < 0.001) and showed adequate two-item reliability (Spearman–Brown coefficient = 0.820); their factor loadings increased substantially, supporting retention of this two-item subscale [25]. Apart from SI1–SI2, the same four item pairs identified in Model 1 received error covariances (Model 2, Version 2). Model 2 showed minimal improvement in fit over Model 1. OH1, SE1, and SE3 had factor loadings below 0.40, and correlations between the SI factor and the other subscales remained low (Fig. 2).

Model 3 was constructed based on EFA cross-loadings and conceptual considerations. OH1 (toothache) was reassigned to the FW domain because it directly affects functional limitations, and FW4 (difficulty speaking) was moved to the SW domain owing to its relevance to communication and socio-emotional outcomes. Error covariances were retained for the same four item pairs (Version 2). Model 3 demonstrated substantially improved fit compared with Models 1 and 2 and yielded the lowest AIC; therefore, Model 3 (Version 2) was selected as the final model.

**Fig. 3:**
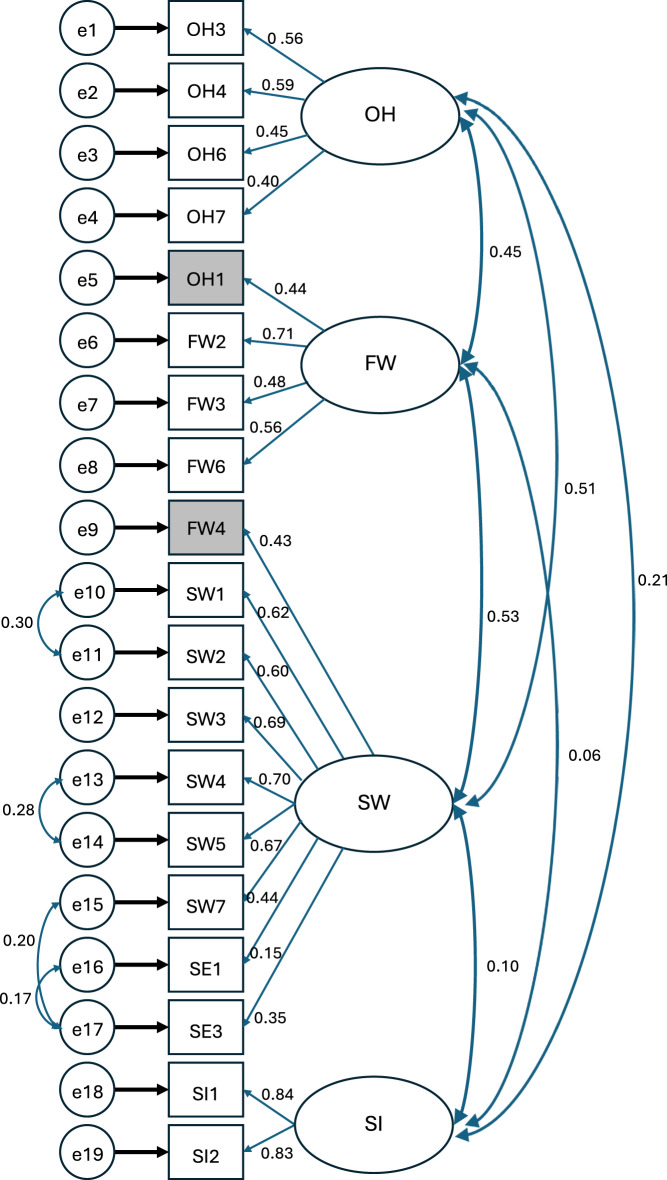
Confirmatory factor analysis of the modified four-subscale COHIP-SF 19 model (final model; Model 3). Two items (OH1 and FW4) are reallocated from Model 2

SE1 and SE3 still had factor loadings below 0.40, and correlations between the SI subscale and the other subscales remained low (Fig. 3). To examine the impact of these items, an additional model excluding SE1 and SE3 was tested (Model 4), which showed slightly improved fit indices compared with Model 3.

**Fig. 4:**
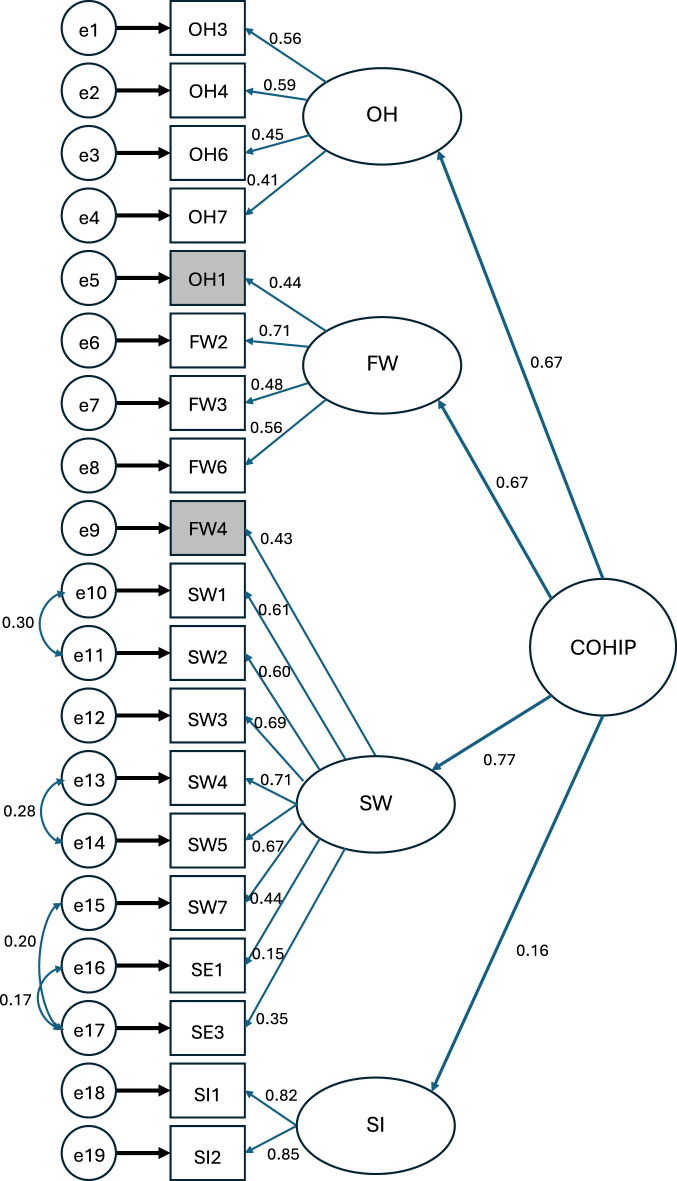
Confirmatory factor analysis of a second-order COHIP-SF 19 incorporating the four-subscale structure of Model 3

A second-order model that added the COHIP-SF 19 total score as a higher-order factor to the final first-order structure with all 19 items (Model 3, Version 2) showed virtually identical fit indices, supporting the use of an overall COHIP-SF 19 score (Table 3, Fig. 4). In this second-order model, standardized path coefficients from the higher-order factor to the four subscales (OH, FW, SW, SI) were 0.67, 0.67, 0.77, and 0.16, respectively.

### Scale-level descriptive statistics

Descriptive statistics for the total score and each subscale in the original and final models (median, IQR, range, and mean ± SD) are presented in Table 4. The total score ranged from 37 to 76 (mean 65.2 ± 5.6).

**Table 4:** Subscale-level descriptive statistics of the Korean version of COHIP-SF 19

		No. of items	Median	IQR	Range	Mean (SD)
Total		19	66	62–69	37–76	65.2 (5.6)
Original model	OH	5	17	15–19	5–20	16.5 (2.7)
	FW	4	16	15–16	7–16	15.4 (1.2)
	SW	10	33	32–36	18–40	33.3 (3.4)
Final model	OH	4	14	12–15	3–16	13.0 (2.5)
	FW	4	16	14–16	7–16	15.0 (1.5)
	SW	9	36	34–36	19–36	34.7 (2.4)
	SI	2	2	0–4	0–8	2.5 (2.3)

OH, oral health; FW, functional well-being; SW, social/emotional well-being; SI, self-image

### Reliability

Test–retest reliability was excellent for the total score (ICC = 0.905, 95% CI 0.863–0.930). In the original model, subscale ICCs (95% CI) were 0.856 (0.799–0.893) for OH, 0.871 (0.854–0.886) for FW, and 0.888 (0.867–0.905) for SW. In the final model, ICCs were 0.856 (0.804–0.891), 0.867 (0.848–0.883), 0.900 (0.880–0.916), and 0.876 (0.859–0.890) for OH, FW, SW, and SI, respectively.

Cronbach’s α for the full 19-item scale was 0.729. Subscale α values were generally higher in the final model than in the original model, except for OH (Table 5). Cronbach’s α-if-item-deleted decreased for all items except SE1 and SI1, for which the increases were negligible. Corrected item-total and item-subscale correlations exceeded 0.20 for all items except SW7, SE1, and SE3.

**Table 5:** Reliability of the Korean version of COHIP-SF 19

		No. of items	Cronbach’s α	Cronbach’s α-if-item-deleted	Corrected item-total score correlation†	Corrected item-subscale score correlation†
Total		19	0.729	0.705–0.734^a^	0.107–0.419^d^	
Original model	OH	5	0.570	0.476–0.564		0.237–0.386
	FW	4	0.562	0.352–0.556		0.225–0.457
	SW	10	0.632	0.584–0.636^b^		0.104–0.502^e^
Final model	OH	4	0.564	0.447–0.536		0.273–0.386
	FW	4	0.580	0.410–0.566		0.288–0.434
	SW	9	0.779	0.727–0.789^c^		0.090–0.562^f^
	SI	2	0.818	N/A‡		0.683

†: Spearman correlation coefficient. All correlation coefficients are significant (*p* < 0.001). ‡Cronbach’s α could not be computed because only one item remained after deletion. Cronbach’s α-if-item-deleted values exceeding the overall α are indicated with superscripts: ^a^ SI1, 0.734; ^b^ SE1, 0.636; ^c^ SE1, 0.789. Correlation coefficients less than 0.20 are indicated with superscripts: ^d^ SW7, 0.193; SE1, 0.107; SE3, 0.159; ^e^ SW7, 0.198; SE1, 0.104; SE3, 0.166; ^f^ SE1, 0.090. OH, oral health; FW, functional well-being; SW, social/emotional well-being; SI, self-image

### Validity

For convergent validity (Table 6), scores in both the original and final models correlated positively with self-rated overall OH and negatively with perceived treatment need. Correlation coefficients were comparable between the two models.

**Table 6:** Convergent validity of the Korean version of COHIP-SF 19 with self-rated oral health and treatment needs

		Overall oral health	Treatment needs
Total score		0.359**	−0.276**
Original model	OH	0.324**	−0.255**
	FW	0.135**	−0.146**
	SW	0.280**	−0.199**
Final model	OH	0.318**	−0.228**
	FW	0.149**	−0.188**
	SW	0.182**	−0.135**
	SI	0.242**	−0.154**

Spearman correlation coefficient. All correlation coefficients are significant (**p < 0.001). OH, oral health; FW, functional well-being; SW, social/emotional well-being; SI, self-image

For discriminant validity (Table 7), children with caries experience or malocclusion had significantly lower total scores, whereas gingivitis and oral hygiene status showed no association. No sex differences emerged in the original model; however, in the final model, girls reported lower scores on the FW and SW subscales than boys.

**Table 7:** Discriminant validity of the Korean version of COHIP-SF 19 based on oral examination findings

			Total score	Original model			Final model			
				OH	FW	SW	OH	FW	SW	SI
		N (%)	Median (IQR)							
df/DMF	0	250 (25.5)	67 (7)	17 (4)	16 (1)	34 (4)	14 (3)	16 (1)	36 (1)	3 (5)
	≥1	730 (74.5)	66 (7)	17 (4)	16 (1)	33 (4)	13 (3)	16 (2)	36 (2)	2 (4)
	*p*-value†		0.005*	0.104	0.337	0.002*	0.108	0.272	0.309	0.002*
Malocclusion	No	695 (70.9)	66 (6)	17 (4)	16 (1)	33 (4)	14 (3)	16 (2)	36 (2)	2 (4)
	Yes	285 (29.1)	65 (8)	17 (4)	16 (1)	32 (5)	13 (4)	16 (2)	36 (2)	2 (4)
	*p*-value†		0.012*	0.056	0.060	0.038*	0.007*	0.788	0.031*	0.258
Gingivitis	No	703 (77.7)	66 (7)	17 (4)	16 (1)	33 (4)	14 (3)	15 (2)	36 (2)	2 (4)
	Yes	277 (26.3)	66 (7)	17 (4)	16 (1)	33 (4)	14 (4)	16 (1)	36 (2)	2 (4)
	*p*-value†		0.423	0.902	0.622	0.337	0.713	0.091	0.402	0.313
Oral hygiene	Good	445 (45.6)	66 (7)	17 (4)	16 (1)	33 (4)	14 (3)	15 (2)	36 (2)	2 (5)
	Moderate	402 (41.2)	66 (7)	17 (4)	16 (1)	33 (3)	14 (3)	15 (1)	36 (2)	2 (4)
	Poor	128 (13.1)	66 (8)	17 (4)	16 (1)	33 (3)	13 (4)	16 (2)	36 (2)	2 (4)
	*p* value‡		0.107	0.754	0.970	0.059	0.599	0.347	0.561	0.100
Sex	Male	508 (51.8)	66 (7)	17 (4)	16 (1)	33 (4)	14 (3)	16 (1)	36 (2)	2 (4)
	Female	472 (48.2)	66 (7)	17 (3)	16 (1)	33 (4)	14 (4)	15 (2)	36 (2)	2 (4)
	*p*-value†		0.542	0.301	0.061	0.589	0.476	0.040*	0.018*	0.069

†Mann–Whitney test, ‡Kruskal–Wallis test, *p < 0.05. OH, oral health; FW, functional well-being; SW, social/emotional well-being; SI, self-image

## Discussion

This study aimed to examine the factor structure of the Korean COHIP-SF 19 in children enrolled in a community-based oral health promotion program and evaluated its reliability and validity. A revised factor structure was identified, and the instrument demonstrated acceptable psychometric properties.

### Contextual considerations and score patterns

The original 34-item COHIP had already been translated and culturally adapted into Korean, and its face and content validity had been confirmed in previous research [7]. These steps were therefore not repeated in the current investigation. Moreover, because the sample consisted exclusively of Korean children residing in Seoul, no comparisons of psychometric properties across ethnic or regional subgroups were conducted.

Total and subscale scores on the COHIP-SF 19 were slightly higher than those reported in earlier validation studies of the same instrument [8, 9, 11–14]. This finding may be attributable to the characteristics of the present community-based sample rather than clinic-based patients, combined with broad access to dental care under Korea’s universal health insurance system and relatively low out-of-pocket costs [26, 27]. Consistent with previous reports [28], a high proportion of children in this study reported negative impacts on self-image items, underscoring the importance of esthetic concerns and self-perception in children’s OHRQoL.

### Evaluation of factor structure

The EFA and CFA results showed that the original three-factor structure of the COHIP-SF 19 demonstrated model fit comparable to previous studies conducted in France and Japan [9, 11]. When the SI items were modeled as an independent factor, factor loadings and internal consistency improved substantially, consistent with findings from the Japanese, French, and Arabic versions [9, 11, 14]. Accordingly, a four-factor structure with SI as a separate domain was proposed, which also aligns conceptually with the original COHIP-34 domains.

The relatively low correlations between the SI subscale and the other subscales were consistent with previous findings [9, 11, 14]. This pattern likely reflects the stronger association of SI items with psychological and identity development in childhood and adolescence rather than with functional or socio-emotional aspects of OH. The relative independence of the SI domain is further supported by the low standardized path coefficient for SI in the second-order model, indicating that the SI subscale captures a distinct dimension within the overall OHRQoL construct measured by the COHIP-SF 19.

Additional item reallocations based on cross-loadings and conceptual considerations yielded a four-factor model with the best overall fit, which was therefore selected as the final structure (Model 3, Version 2). The reassignment of OH1 to the FW subscale and FW4 to the SW subscale aligns with findings from Skandrani et al. [9]. However, the specific items reassigned and the resulting factor configurations have varied across studies, indicating that the COHIP-SF 19 does not exhibit a universally consistent structure and that factor loadings for specific items may differ depending on cultural and linguistic context [9, 14, 29]. These findings highlight the need for culture-specific validation and support presenting results from both the original and final models to facilitate comparison with previous literature. The second-order model exhibited fit indices comparable to those of the final four-factor first-order model, confirming the structural appropriateness of the COHIP-SF 19 total score. This finding provides the first empirical evidence for the structural validity of the total score use in the COHIP-SF 19.

### Reliability and validity

The Korean COHIP-SF 19 showed excellent test–retest reliability for both the total and subscale scores, indicating good measurement stability over time. Cronbach’s α for all 19 items was 0.729, which reflects satisfactory internal consistency [22]. Although the subscale-level alphas were modest, they were similar to those reported in previous studies [9, 11, 12] and are consistent with the well-known tendency for Cronbach’s α to be lower when subscales contain few items [22]. The final four-factor model generally produced higher α values than the original three-factor model; the slight decrease observed for the OH subscale can be explained by the reduction from five to four items [22].

Items SE1 and SE3 showed low factor loadings, and model fit improved slightly when these items were removed (Model 4). However, the model retaining all 19 items (Model 3) was selected as the final model to preserve comparability with previous COHIP-SF 19 studies and to maintain the content validity of the original instrument. In addition, SW7, SE1, and SE3 displayed somewhat lower item-total correlations, possibly because of restricted response variability in this community-based sample, wherein caries and malocclusion were relatively uncommon. Future research involving clinical samples with more varied oral conditions is needed to further evaluate the suitability of these items.

Convergent validity was supported in both the original and final models, as COHIP-SF 19 scores correlated positively with self-rated oral health and negatively with perceived treatment need. Discriminant validity was also confirmed: children with dental caries experience or malocclusion had lower total scores, consistent with findings from earlier studies in other cultural settings [9, 11, 12, 14]. These results indicate that the Korean version of the COHIP-SF 19 possesses adequate convergent and discriminant validity, particularly in relation to dental caries and malocclusion.

However, gingivitis and oral hygiene status were not associated with total scores. This pattern aligns with previous research conducted among urban children [11], where the prevalence of gingivitis was low and oral hygiene tended to be good. It is also possible that children are less aware of gingival health or oral hygiene than of dental caries or malocclusion, which may contribute to the weaker associations observed in this study [30, 31].

No significant sex differences were observed in the total score or the subscales of the original model. However, in the final model, girls showed reduced OHRQoL in the FW and SW subscales, suggesting that the revised structure may be more sensitive to detecting sex-related differences. Research using the French and Arabic versions of the COHIP-SF 19 reported similar findings [9, 14], reflecting the more significant physical changes and heightened psychological sensitivity experienced by girls during development [32]. Conversely, several studies report higher OHRQoL among girls [7, 11, 12]. These discrepancies suggest that sex-related differences in OHRQoL may vary according to cultural context, the factor structure of the instrument, and the age distribution of the study population [32].

### Strengths and limitations

This study represents the first psychometric validation of the COHIP-SF 19 in Korea, providing culturally relevant evidence for its use among Korean children. The large community-based sample also enabled a comprehensive evaluation of structural, convergent, and discriminant validity. However, the participants were limited to 10-year-old elementary school students residing in Seoul, and the recruitment process was not based on random sampling. Although participants were drawn from all 25 administrative districts in Seoul, the sample was not designed to be representative of the population. Consequently, the findings cannot be generalized to entire Korean child population. Future studies should examine structural validity in samples covering broader age ranges, geographic regions, and socioeconomic backgrounds. Because both the EFA and CFA were conducted within a single sample, cross-validation using an independent sample is needed. Although all 19 items were included in the present analysis, future development of shortened versions may consider removing or replacing items such as SE1, SE3, and SW7.

## Conclusion

This study demonstrated that the Korean version of the COHIP-SF 19 has satisfactory psychometric properties for assessing OHRQoL in Korean schoolchildren. EFA and CFA revealed a modified four-factor model that separates the SI subscale and offers better structural validity and higher internal consistency than the original three-factor structure. The modified model also provided clearer subgroup discrimination, including sex differences, thereby supporting its use in clinical and epidemiological settings. Further studies with more diverse age groups and populations are required to confirm the stability and generalizability of the proposed model.

## Data Availability

Data supporting the findings of this study are available from the corresponding author on reasonable request. The data are not publicly available owing to privacy and ethical restrictions.

## Abbreviations

AIC: Akaike information criterion
COHIP-SF 19: Child Oral Health Impact Profile Short Form 19
CFA: Confirmatory factor analysis
CFI: Comparative fit index
χ^2^/df: Chi-square minimum discrepancy divided by degrees of freedom
EFA: Exploratory factor analysis
FW: Functional well-being
ICC: Intraclass correlation coefficient
MI: Modification index
OH: Oral health well-being
OHRQoL: Oral health-related quality of life
RMSEA: Root mean square error of approximation
SDHP: Student dental home program
SE: School environment
SI: Self-image
SRMR: Standardized root mean square residual
SW: Social/emotional well-being
TLI: Tucker–Lewis index
